# *In vitro* modeling of glucocorticoid mechanisms in stress-related mental disorders: Current challenges and future perspectives

**DOI:** 10.3389/fcell.2022.1046357

**Published:** 2022-11-28

**Authors:** Katherine Bassil, Laurence De Nijs, Bart P. F. Rutten, Daniel L. A. Van Den Hove, Gunter Kenis

**Affiliations:** Department of Psychiatry and Neuropsychology, School for Mental Health and Neuroscience (MHeNs), Faculty of Health, Medicine and Life Sciences, Maastricht University, Maastricht, Netherlands

**Keywords:** stress, glucocorticoids, neurons, *in vitro*, neuropsychiatry

## Abstract

In the last decade, *in vitro* models has been attracting a great deal of attention for the investigation of a number of mechanisms underlying neurological and mental disorders, including stress-related disorders, for which human brain material has rarely been available. Neuronal cultures have been extensively used to investigate the neurobiological effects of stress hormones, in particular glucocorticoids. Despite great advancements in this area, several challenges and limitations of studies attempting to model and investigate stress-related mechanisms *in vitro* exist. Such experiments often come along with non-standardized definitions stress paradigms *in vitro*, variations in cell models and cell types investigated, protocols with differing glucocorticoid concentrations and exposure times, and variability in the assessment of glucocorticoid-induced phenotypes, among others. Hence, drawing consensus conclusions from *in-vitro* stress studies is challenging. Addressing these limitations and aligning methodological aspects will be the first step towards an improved and standardized way of conducting *in vitro* studies into stress-related disorders, and is indispensable to reach the full potential of *in vitro* neuronal models. Here, we consider the most important challenges that need to be overcome and provide initial guidelines to achieve improved use of *in vitro* neuronal models for investigating mechanisms underlying the development of stress-related mental disorders.

## 1 Introduction

**FIGURE 1:**
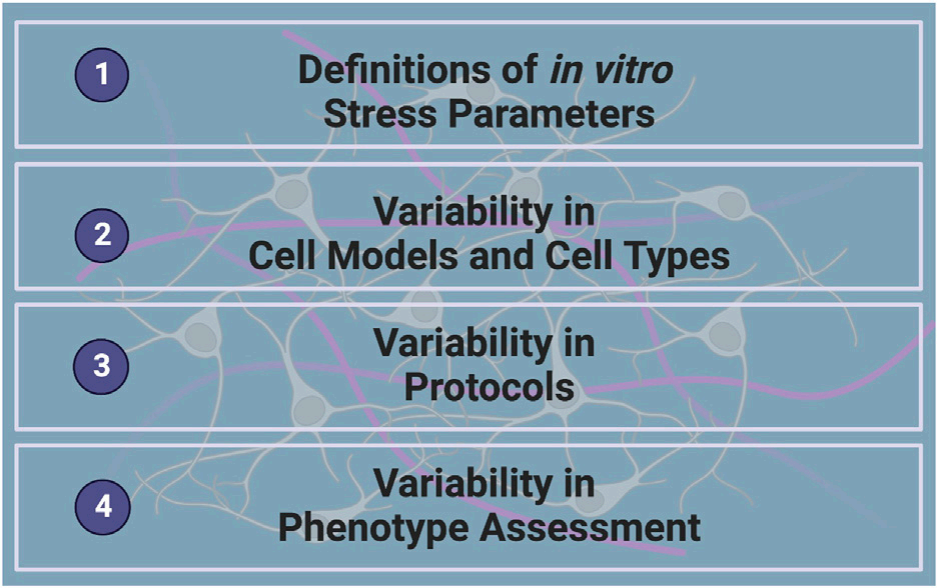
Challenges in Investigating the Neurobiological Effects of GCs *in vitro*.

Modeling stress and its effects has long been conducted in animal models, with different stress models highlighting different stress mechanisms and processes (e.g., resilience versus susceptibility) (Sutanto and De Kloet, 1994; Franklin et al., 2012). *In vitro* models for stress-related mental disorders (SRMDs), allow the investigation of the effects of key stress hormones [namely glucocorticoids (GCs), norepinephrine, etc.]—independently or in combination—on cellular [e.g., neurogenesis (Schoenfeld and Cameron, 2015)], molecular, and (electro) physiological processes hypothesized to be involved in SRMDs, and more recently on regulation of disorder-specific genetic variants [e.g., *FKBP5* (Smoller, 2016)]. Additionally, *in vitro* models are relatively cost and time-efficient, and overcome many of the ethical considerations associated with using research animals (Graudejus et al., 2018), especially with the discovery of cellular programming and reprogramming technology (CPART)—namely the generation of induced pluripotent stem cells (iPSCs) from adult human somatic cells (Marchetto and Gage, 2012). Investigating stress mechanisms *in vitro* (as most molecular biology assays) is a highly reductionist approach (Regenmortel, 2004) to understanding stress, its underlying processes, and the mechanisms of SRMDs more broadly. That being said, *in vitro* stress models aim to investigate underlying mechanisms involved in the stress response, as a reaction to exposure to particular stress hormones, with the most studied hormone being GCs (Bhargava et al., 2002; Kim et al., 2004; Cote-Vélez et al., 2005; Numakawa et al., 2009; Anacker et al., 2013; Bharti et al., 2018; Nürnberg et al., 2018; Yeo et al., 2019; Krontira et al., 2020; Cruceanu et al., 2021; Heard et al., 2021; Choi et al., 2022). In essence, one would assume that investigating effects of GCs *in vitro* seems straightforward. However, the literature shows that GC-induced responses in cultured cells are influenced by many factors, which severely impedes drawing clear, unequivocal conclusions. We believe that increasing the level of standardization in these studies is essential to ensure reproducibility and increased validity of *in vitro* models. It should be acknowledged however, that every experimental setup and design is in fact research question-dependent and as such may require different approaches and conditions. In this perspective, we highlight some of the challenges in investigating the effects of stress hormones in different *in vitro* models by using GCs as an example (Figure 1), and formulate recommendations for improvement.

## 2 The state-of-the-art

### 2.1 Modeling aspects of stress *in vitro*

**FIGURE 2:**
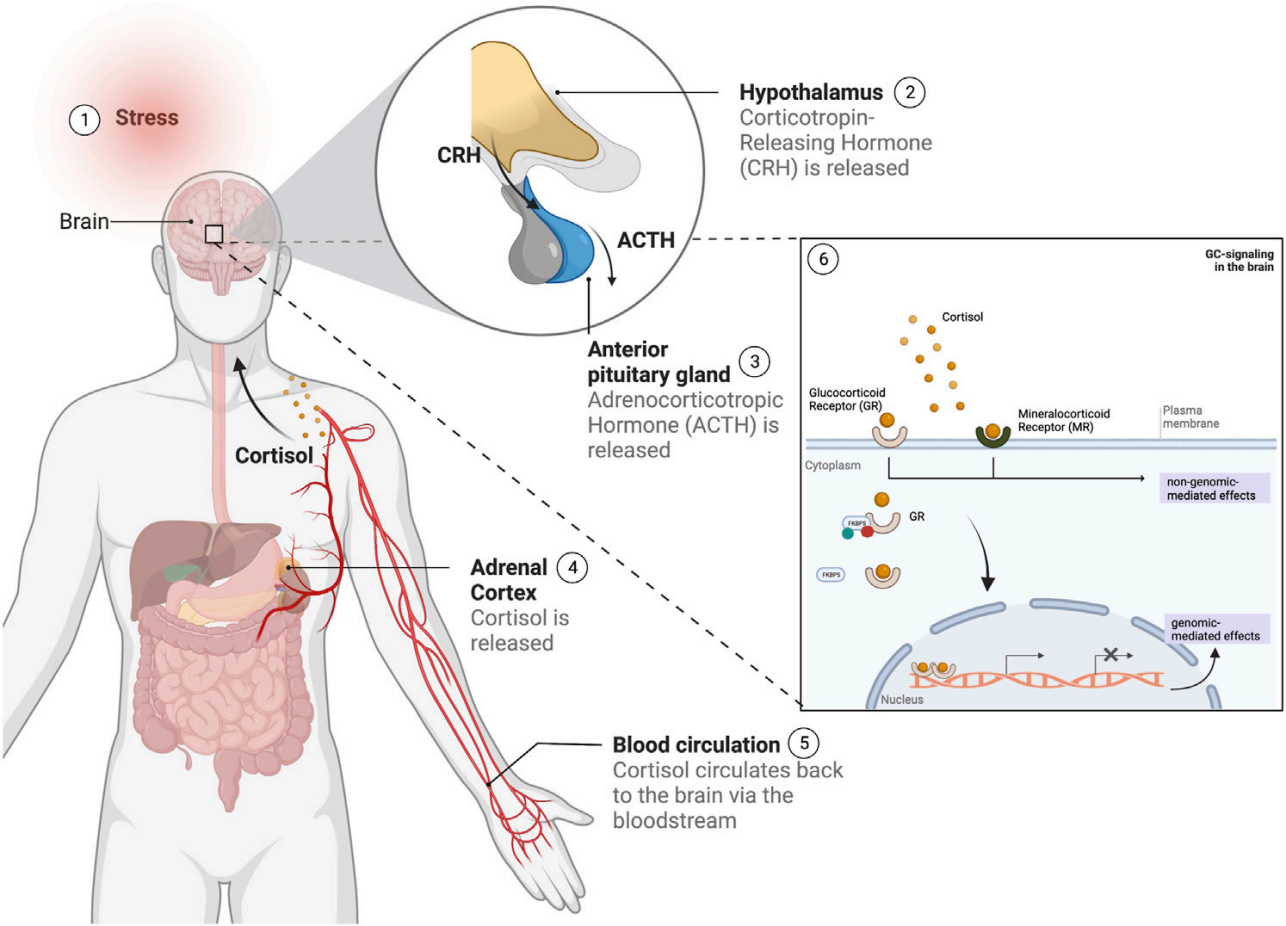
Overview of the stress response in humans following a stress stimulus. 1) Upon the experience of a stress stimulus, 2) the hypothalamus is activated and releases 'corticotropin-releasing hormone (CRH), which leads to the 3) activation of the anterior pituitary gland to secrete adrenocorticotropic hormone (ACTH) in the blood stream, followed by 4) the stimulation of the adrenal cortex to release the glucocorticoid cortisol. Cortisol 5) is circulated *via* the bloodstream to the brain, where 6) it will bind to the glucocorticoid and mineralocorticoid receptors (GR and MR respectively), regulated by gene regulators such as the *FKBP5* and other co-factors, and whose activation will lead to a number of signaling cascades leading to both genomic and non-genomic-mediated effects. (This figure was created with BioRender).

The neurobiology of stress encompasses a number of mechanisms, including the activation of the autonomic nervous system and the hypothalamic-pituitary adrenal (HPA) axis, with each involving different hormones and regulators such as (nor) adrenaline, corticotropin-releasing hormone (CRH), andrenocorticotropic hormone and GCs (Johnson et al., 1992) (Figure 2). Together these mechanisms work in concert to enable an individual to respond to stressors (of an acute or chronic nature) and bring the systems back to homeostasis (Sutanto and De Kloet, 1994). Dysregulation of the HPA axis, more specifically an impairment in its negative feedback regulation, has been involved in a number of SRMDs including major depressive disorder (MDD) and post-traumatic stress disorder (PTSD) (Yehuda, 2002). Loss of negative feedback leads to HPA-axis hyperactivity in MDD, while the reverse is observed in PTSD resulting in hyporesponsivity of the HPA axis (Yehuda, 2002). Prolonged exposure to GCs as a consequence of chronic or repeated stress experiences, has neurotoxic effects which induce several metabolic and cellular vulnerabilities, and which are believed to underlie causative factors in the onset and development of SRMDs (Sapolsky, 1996; Popoli et al., 2012; Chattarji et al., 2015; McEwen et al., 2016).

GCs such as cortisol (in humans) and corticosterone (in rodents) mediate their effects *via* two receptors: the glucocorticoid receptors (GR) and the mineralocorticoid receptors (MRs), with the MR showing higher affinity for GCs than the GR (Koning et al., 2019). An imbalance in GR- and MR-mediated responses are thought to increase risk for SRMDs (De Kloet and Meijer, 2019). While MR dysregulation has also been reported in SRMDs (De Kloet et al., 2016), the detrimental effects of GCs are predominantly ascribed to GR-mediated signaling, and hence most *in vitro* GC studies focus on the downstream effects of GR activation. This has improved our knowledge on the effects of GCs on several neuronal processes including neurogenesis, neuronal morphology, synaptogenesis, and synaptic plasticity, among others (Finsterwald and Alberini, 2014), and have helped us better understand the involvement of GCs in SRMDs (Xi et al., 2011; Lieberman et al., 2017; Seah et al., 2021).

Recent developments in the field of CPART make the use of *in vitro* stress models even more relevant (Marchetto and Gage, 2012), since it allows to investigate GC-induced cellular responses in the context of the genetic background of individuals expressing differential susceptibility to develop SRMDs. Indeed, the identification of unique gene expression signatures and related pathways implicated in stress vulnerability, have been identified in neurons and glia from iPSCs derived from SRMD patients (Vadodaria et al., 2019a; Vadodaria et al., 2019b; Vadodaria et al., 2019c; Seah et al., 2021; Heard et al., 2021). These models can also be used to examine the effects of genetic risk variants of SRMDs, e.g., polymorphisms in *NR3C1* (the gene coding for GR) (Peng et al., 2018), or differential responses to GC exposure between iPSC-derived neurons from healthy and SRMD patients (Seah et al., 2021; Heard et al., 2021). The investigation of the neurobiological effects (at the molecular, cellular, morphological, and physiological levels) of hormones (including cortisol), drugs (incl. antidepressants), or other molecules of interest separately or in combination with one another [e.g., cortisol and (nor) epinephrine; or cortisol and antidepressants] can be performed *in vitro* in a highly controlled environment, without the interference of other systems and molecules. The effects of drugs, hormones, and other molecules on a certain type of neuron implicated in SRMDs (e.g. serotoninergic neurons in MDD; cortical neurons in PTSD), can also be investigated through CPART (Marchetto and Gage, 2012). Moreover, this technology allows the investigation of pathways and connections between two distinct types of cells (e.g., between different types of neurons or between neurons and glia) in the form of co-cultures [for an example, see (Hedegaard et al., 2020)]. CPART has also enabled the study of the effects of drugs, hormones, or molecules in human cerebral organoids (Cruceanu et al., 2021)—3D *in vitro* models of neuronal development, with distinct cellular responses in different types of neural progenitor cells, and neurons. Furthermore, the *in vitro* manipulation of genetic variants (e.g., single nucleotide polymorphisms) or epigenetic mechanisms (DNA methylation of key genes in the stress response, such as *GR* and *FKBP5*) using, for example, recent cutting-edge technology such as CRISPR-cas9, is highly desirable and in many cases more efficient. Finally, *in vitro* studies can be used to model aspects of hypo- or hyper-suppression of the HPA axis, by for instance the manipulation of the GR receptors and sensitivity using agonists and antagonists, in the presence of GCs at different concentrations.

Provided that more reliable and standardized protocols for investigating aspects of stress *in vitro* exist, this may bring about major advances in the areas of stress susceptibility and resilience. *In vitro* modeling could serve as a tool to investigate potential drugs for SRMDs prior to testing on patients, and identify novel target mechanisms, candidate genes, and neuronal subtypes involved. In addition, *in vitro* models may be pivotal as a personalized medicine approach (among others) for SRMD patients (Marchetto and Gage, 2012). To harness the full potential of *in vitro* models, more complex experimental designs may need to be introduced, such as going from examining the neurobiological effect of only one hormone to a combination of stress mediators, and in defined temporal sequences. Obviously, some degree of standardization in this respect would help in moving the field forward.

### 2.2 *In vitro* options for investigating the neurobiology of glucocorticoids

Based on the source of the cells being used, there are a few major groups of *in vitro* techniques that have been used to investigate the neurobiological effects of GCs. In the literature, the majority of *in vitro* studies investigating GC effects are performed on 2D neuronal cultures, which can grossly be categorized in three methodologies. The first and oldest technique is the use of animal primary neural progenitor or neuronal cells harvested from different brain regions (Zhou et al., 2012; Zhu et al., 2018). The second corresponds to neuroblastoma cell lines (human or animal immortalized cells that can be differentiated in a neuron-like phenotype) and include cell lines such as the human SH-SY5Y cells (Cote-Vélez et al., 2005; Wong et al., 2015; Sabbagh et al., 2018; Liu et al., 2021). The third group entails embryonic stem cell (ESC)—or iPSC-derived heterogeneous neuronal cultures (Ninomiya et al., 2014; Raciti et al., 2016; Nürnberg et al., 2018; Yeo et al., 2019). This category also includes the direct conversion (or trans-differentiation) of adult somatic cells into neuronal cultures (Seah et al., 2021). Beyond 2D cultures, the generation of 3D brain models such as cerebral organoids and assembloids has recently gained significant interest (Di Lullo and Kriegstein, 2017). Cerebral organoids can also be generated from ESC or patient-derived iPSCs, are characterized by more relevant heterogeneity of cell types, and capture to some extent the cytoarchitecture of the human brain (Shou et al., 2020). Each method carries its own advantages and limitations with some being mentioned in this review (Gordon and Geschwind, 2020).

## 3 Beyond the challenges

In order to reach the full potential of *in vitro* models in understanding the underlying mechanisms of SRMDs, some important challenges related to investigating the neurobiological effects of GCs *in vitro* must first be overcome. This includes defining *in vitro* stress parameters, identifying and tackling sources of variability in cell models, culture and differentiation protocols and molecular or cellular readouts (Figure 1). Improving *in vitro* GC studies will heavily rely on the development of more standardized protocols and methodologies specific to neuronal cultures and the unique research question, in a way that is not only standardized but also reproducible.

### 3.1 Defining stress parameters *in vitro*

To successfully model aspects of stress mechanisms *in vitro*, an approach that first deals with the semantics of stress and defining certain stress parameters *in vitro*, might be favorable. First it is necessary to define what we mean by *in vitro* models of SRMDs. In the literature, models of stress *in vitro* can refer to metabolic, oxidative, or mechanical stress models (Garcia et al., 2015; Abdullahi et al., 2017; Choi et al., 2021). However, in the context of SRMDs, an *in vitro* model of stress usually refers to the exposure of a neuronal culture to GCs -, e.g., cortisol or synthetic agonists of GC receptors such as dexamethasone, (nor) adrenaline, and/or other mediators of the *in vivo* stress response. Just like animal models of stress, *in vitro* exposure to a chemical stressor can be acute or chronic (Sheridan et al., 2004). While acute stress represents exposure to stress for a relatively short amount of time, chronic stress reflects repetitive and/or prolonged stress exposure (Sheridan et al., 2004). Moreover, the effects of (both acute or chronic) stress can be studied shortly after the exposure or after a delayed period of time. In general, there is a lack of consensus as to what defines acute and chronic stress and what defines short-term versus long-term effects. In our opinion, these are important parameters that need to be clearly defined in order to create standardized protocols that can be reproducible and to obtain better *in vitro* model systems to study stress-related mechanisms (Richter-Levin and Sandi, 2021). Additionally, defining acute and chronic stress might even allow to model and investigate the concepts and molecular mechanisms of allostasis and allostatic load *in vitro* (McEwen, 1998), as suggested by McEwen (McEwen, 2002) on the use of cultures to examine hormonal interactions, such as mechanisms in allostasis. For instance, acute stress in *vivo* models is seen as a single exposure to a stimulus that initiates a stress response, and of which the cellular and molecular effects in the brain can be short- or long-lasting (Musazzi et al., 2017). Consequently, an acute *in vitro* GC challenge could be defined as a single exposure to GCs for a short period of time. Defining the latter is difficult as it is unclear how the *in vitro* kinetics and signaling of GCs relate to *in vivo* conditions (a common challenge in cell culture models). In current literature, typical acute exposure times range from hours to 48 h, which makes it virtually impossible to draw unifying conclusions. In contrast, congruent to *in vivo* conditions, a chronic *in vitro* GC challenge could be understood as a repetitive and prolonged exposure to GCs (e.g., ranging from days to weeks) with a GC-induced phenotype persisting for more than a few days (e.g., more than 72 h). While it is difficult to setup specific guidelines as to what constitutes an acute and chronic exposures, providing clear descriptions and harmonization of paradigms, will benefit the field to increase the reproducibility of *in vitro* protocols and results.

### 3.2 Sources of variability

#### 3.2.1 Cell models and cell types

A number of different brain cell models have been employed to investigate the effects of GCs on neurons and on different types of glial cells. These included primary cultures, immortalized cell lines, pluripotent stem cell-derived neuronal cultures (2D and 3D), and different types of glial cells, among others. Different cultures introduce a number of variations, first due to the nature of the cell source and, second, related to the different culture mediums used for each culture types, with different supplements including serum. It is important to mention that the presence of GCs in neuronal differentiation media is necessary to drive differentiation *in vitro* (Odaka et al., 2017), which could lead to interferences in assessing the neurobiological effects of GCs added to a culture. Moreover, many of the differentiation protocols to obtain neuronal cultures are heterogeneous in cell types and many include glial cells (Volpato and Webber, 2020). This may in itself influence the response of neurons to GCs knowing that glial cells such as astrocytes have also been shown to respond to GCs *in vitro* (Heard et al., 2021; Choi et al., 2022). Additionally, a study by Cruceanu et al. (2022) demonstrated cell-type specific responses to GCs *in vitro*, with differential-responses between different types of neural progenitors and neurons (Notaras et al., 2021). Current studies are mainly performed on heterogeneous cultures of neurons, glia, and non-neuronal cells. Future studies should better investigate GC-induced effects in pure neuronal and pure glial cultures (by making use of CPART) such as to facilitate drawing conclusions on the effects of GCs on neurons alone, on neuronal-glial co-cultures, and/or neuronal-glial co-cultures with glial cells pre-treated with GCs. Investigating GC effects in pure cultures alone will allow us to better understand the effects of GCs on distinct cell types that might have a key role in the pathophysiology of certain SRMDs (e.g., serotonergic neurons in MDD). Whereas co-cultures have the advantage of allowing us to investigate the interaction between neurons and glial for example, which more closely resembles *in vivo* processes in normal and pathological conditions.

Important, yet often overlooked parameters, when investigating GC effects *in vitro* are GC receptor expression and GC sensitivity in the examined cells. GC receptor mediated responses are influenced by GC receptor expression on the one hand and sensitivity of the downstream signaling cascades mediated by chaperone and other interacting signaling molecules. Knowing that GR and MR expression differ *in vivo* and *in vitro*, studies should consider expression levels and their ratios of the two receptors in the different cell lines, and results should be interpreted in that context.

For example, Lieberman et al. (2017) investigated GC vulnerability in iPSC-derived forebrain neurons from patients carrying an *FKBP5* risk variant and found no effects of dexamethasone, a selective GR agonist, on GR expression in at risk carriers. Their results suggest that low expression of GR in stem cell-derived neurons with a maturation state comparable to fetal neurons (Marchetto and Gage, 2012) might prove challenging to investigate some GC-induced phenotypes. Nevertheless, despite observing no significant changes on neuronal processes such as proliferation and differentiation, GCs may still have an effect on other outcome parameters, and one should be aware of the limitations that the different cell lines carry (i.e., GC receptor levels) which should be considered in the design and setup of their experiments. Advancements in stem cell differentiation protocols and techniques might 1 day improve the phenotype of the generated neurons and hence improve sensitivity of neuronal cell lines to GCs by expressing GR and MR levels more representative of *in vivo* conditions.

Further, understanding the effects of GC signaling *via* these receptors separately is essential. GC-induced signaling *via* GR and MR has differential effects which can be examined using selective agonists and antagonists of each receptor (McMaster and Ray, 2008). Understanding these differences may help when comparing cell cultures that differ in GR/MR expression levels. On the other hand, in *vivo* conditions both receptors work in concert to establish the overall effect of GCs. Studies using endogenous GCs (i.e., cortisol or corticosterone) could be more informative in that respect. Investigators should carefully consider the type of GC to use, and should clearly indicate the rationale in future publications.

#### 3.2.2 Protocols

One source of variability–and a big limitation of *in vitro* studies in general–is experimental variability between different batches of the same cell line (Burroughs et al., 2012; Marchetto and Gage, 2012), or batch-to-batch variability. Another source of variability concerns the face validity of cell lines. For example, neuroblastoma cell lines carry cancerous properties and as such do not reflect the normal growth and differentiation of neurons in culture (Xicoy et al., 2017). Neuroblastoma cells also carry major limitations in their differentiation potential and maturation state. Additionally, rodent primary neuronal cultures can answer a limited number of research questions given their predetermined fate upon harvest.

One way to address these variabilities is to move away from using unreliable cell lines and more towards improved cell models. For instance, patient-derived neuronal cultures have the advantage of investigating genetic-exposure interactions in different possible neuronal identities and in other cell types. Despite several advantages, stem cell technology also suffers from variability in protocols. The use of different protocols to generate 1) PSC-derived neuronal or glial cultures, including the direct and indirect method, also bring about increased variations (Marchetto and Gage, 2012). For example, Seah et al. (2021) observe differential responses to GCs between induced-neurons and iPSC-derived neurons (Marchetto and Gage, 2012). However, for improved representation of the effects of GCs in humans, one might want to focus on making use of reprogrammed cell lines, and explore the effects of GCs in different neuronal cell types. With the use of reprogrammed cells, individual genomic variation among patients with different genetic background introduces additional variability in the response of neuronal cultures to GC challenges, which should be addressed by using a sufficient number of control- and patient-derived cell lines. Alternatively, the use of isogenic lines could be used to examine the influence of specific genetic variants in relation to GC responses. With batch-to-batch variability being an issue, one need not focus on the use of 1 cell line only, but instead one could focus the bulk experiments on the most robust cell line, and use other cell lines as validation. Acknowledging the advantages and disadvantages of each model in the initial phases of research design is important in overcoming many of these roadblocks, and in improving standardization of *in vitro* studies.

In addition, a systematic overview of convergent evidence from both animal and *in vitro* models could help identify reliable approaches for investigating GC effects on (non-)neuronal cultures and facilitate a better understanding of different protocols employed, promote exchange of methodologies, and improve standardization.

#### 3.2.3 Assessing glucocorticoid-induced phenotypes *in vitro*

While the type of *in vitro* model and the hormone to be investigated are important choices to be made during the design of a study, another challenge is the assessment of the GC-induced phenotype *in vitro* in acute or chronic conditions.

Several readouts have been considered for the detection of a GC-responsive culture such as cytotoxicity and proliferative assays–namely the 2,5-diphenyl-2H-tetrazolium bromide (MTT) assay—but this might not be sufficient. The selection of this readout as an assessment of a GC-induced phenotype is based on *in vivo* studies where increased corticosterone levels lead to cell death and a decrease in proliferation of neuronal cell populations (Abdanipour et al., 2015). While these cellular processes explain some of the effects of GCs *in vitro*, they do not assess the full scale of possible GC-induced phenotypes. The colorimetric MTT-assay–most often used as an assay to measure cellular metabolic activity–has also been used in a number of studies as a readout to test different GC concentrations. While a metabolic assay is important in identifying GC effects in cell cultures, it is not reliable as an accurate measurement of cell viability or cytotoxicity and hence has questionable value as a standardized readout for GC effects (Ghasemi et al., 2021). It has been reported that the MTT assay suffers from a number of limitations in the interpretation of cell viability and cytotoxicity measures [for an extensive explanation, see (Ghasemi et al., 2021)], and as such the results of such a colorimetric assay should be followed by complementary assays. While broadly used as a readout to assess effects of different concentrations of a drug and specifically in neuroprotection studies seeking to reverse the negative effects of GCs, its value in assessing neuronal cultures is now questioned, and, instead, flow cytometry assays for cell viability and toxicity are suggested (Burroughs et al., 2012). Moreover, there are doubts whether using MTT assays for assessing the effects of GCs is the best approach in terms of the pathophysiological context of SRMDs, given that cell death is not the major cause of hippocampal atrophy in SRMDs such as MDD and PTSD (Bremner, 1999; Sapolsky, 2000). For instance, looking into neuronal-specific readouts such as neuronal morphology that relate to, e.g., atrophy such as soma size, neurite length, branching and complexity, or even neuronal live-imaging might be preferred means to assess direct GC-effects. That being said, neuronal subtype (i.e., cortical versus hippocampal), research question, and disease etiology or symptomatology, should all be taken into account and used as a justification for performing MTT assays. For a better assessment of GC-induced phenotypes *in vitro*, some groups have looked instead at the expression levels of a few known glucocorticoid-response element (GRE) containing genes such as *FKBP5*, *TSC22D3*, and *SGK1* (Heard et al., 2021; Cruceanu et al., 2022), which is an improved method in showcasing many of the changes seen following a GC challenge. Moreover, there is an increase in transcriptomic and epigenetic studies (single-cell and bulk) of *in vitro* neuronal cultures following exposure to GCs (Provençal et al., 2019; Seah et al., 2021), which may help define hallmarks to assess GC-induced phenotypes in neuronal cultures in the future. It is important to keep in mind, that different *in vitro* models and GC concentrations may bring about different outcomes and will hence make it more challenging to generalize.

Another variation among *in vitro* studies is the wide range of GC concentrations being tested, which hampers drawing solid conclusions from studies presenting contradictory results. This specific concern could be addressed by developing more stringent methodologies for selecting a concentration range that best resembles *in vivo* healthy and non-healthy conditions. In the literature, there has been no attempt to define general criteria for an acceptable concentration range of GCs to be tested, however if we wish to produce standardized and reproducible *in vitro* studies, more research into the influence of different GC concentrations in different cell lines is needed to reduce sources of variability and better model GC effects *in vitro*. It is important to note, that *in vitro* GC concentrations used are relatively much higher than the possible levels in individuals following a stressful experience or in SRMD patients (Anacker et al., 2013). However, this increased concentration can be justified by the nature of the medium being used, and the presence of certain molecules that breakdown the availability of GCs in culture, hence requiring higher concentrations to reach the required effect (Anacker et al., 2013). That being said, *in vivo* physiological concentrations might not be a good reference.

Overall, the use of relevant GC-induced phenotypes is important and may depend on the research question at hand. Viability assays, despite being commonly used, are not sufficient to evaluate the effects of GC exposure and should be complemented with expression levels of GC responsive genes and proteins, and/or with measures of neuronal morphology relevant to SRMDs. While it is clear that this is a challenge in and of itself, standardized measurements such as expression of GRE-containing genes, multi-omic data, and using various assessments of cellular morphology to test for concentration ranges of GCs and their effects in central nervous system cells are recommended approaches.

## 4 Conclusion

Recent developments instigated progress in modeling stress in-a-dish, although many challenges remain on the road ahead. While many of the challenges may be technical in nature, several equally important ones are more fundamental, especially when it comes to defining stress parameters *in vitro* and selecting the most suited cellular model(s).

It is therefore important to provide sufficient background information and to describe in detail the reasoning behind the selection of a particular cellular model, the type of GC employed, the concentration and exposure time, and the GC-induced phenotype. In addition, authors should be critical of their choices and describe the advantages and limitations of their model, in order for future studies to be improved. Eventually, we foresee that the optimal range of GC concentrations, and criteria for acute and chronic *in vitro* exposures for particular research questions will need to be clearly specified and used across laboratories. Along similar lines, the implementation of robust and more harmonized assessments of GC-induced phenotypes is necessary.

*In* order to allow for *in vitro* studies to fulfill their full-fledged potential and improve our understanding of stress-related mechanisms in health and disease, it is imperative to tackle these issues. Nevertheless, the invested effort will help in identifying the exact underlying mechanisms contributing to stress susceptibility and resilience, increase our understanding of SRMDs, and may finally lead to new therapeutic strategies.

## References

[B1] Abdanipour A. Sagha M. Noori-Zadeh A. Pakzad I. Tiraihi T. (2015). *In vitro* study of the long-term cortisol treatment effects on the growth rate and proliferation of the neural stem/precursor cells. Neurol. Res. 37 (2), 117–124. 10.1179/1743132814Y.0000000431 25082549

[B2] Abdullahi A. Stanojcic M. Parousis A. Patsouris D. Jeschke M. G. (2017). Modeling acute ER stress *in vivo* and *in vitro* . Shock (Augusta, Ga.) 47 (4), 506–513. 10.1097/SHK.0000000000000759 27755507 PMC5348263

[B3] Anacker C. Cattaneo A. Luoni A. Musaelyan K. Zunszain P. A. Milanesi E. (2013). Glucocorticoid-related molecular signaling pathways regulating hippocampal neurogenesis. Neuropsychopharmacology 38 (5), 872–883. 10.1038/npp.2012.253 23303060 PMC3672002

[B4] Bhargava A. Mathias R. S. McCormick J. A. Dallman M. F. Pearce D. (2002). Glucocorticoids prolong Ca2+ transients in hippocampal-derived H19-7 neurons by repressing the plasma membrane Ca2+-ATPase-1. Mol. Endocrinol. 16 (7), 1629–1637. 10.1210/mend.16.7.0861 12089356

[B5] Bharti V. Tan H. Chow D. Wang Y. Nagakannan P. Eftekharpour E. (2018). Glucocorticoid upregulates thioredoxin-interacting protein in cultured neuronal cells. Neuroscience 384, 375–383. 10.1016/j.neuroscience.2018.06.001 29894818

[B7] Bremner J. D. (1999). Does stress damage the brain? Biol. Psychiatry 45 (7), 797–805. 10.1016/s0006-3223(99)00009-8 10202566

[B8] Burroughs S. L. Duncan R. S. Rayudu P. Kandula P. Payne A. J. Clark J. L. (2012). Plate reader-based assays for measuring cell viability, neuroprotection and calcium in primary neuronal cultures. J. Neurosci. Methods 203 (1), 141–145. 10.1016/j.jneumeth.2011.09.007 21968036 PMC3221776

[B9] Chattarji S. Tomar A. Suvrathan A. Ghosh S. Rahman M. M. (2015). Neighborhood matters: Divergent patterns of stress-induced plasticity across the brain. Nat. Neurosci. 18 (10), 1364–1375. 10.1038/nn.4115 26404711

[B10] Choi G. E. Chae C. W. Park M. R. Yoon J. H. Jung Y. H. Lee H. J. (2022). Prenatal glucocorticoid exposure selectively impairs neuroligin 1-dependent neurogenesis by suppressing astrocytic FGF2–neuronal FGFR1 axis. Cell. Mol. Life Sci. 79 (6), 294–323. 10.1007/s00018-022-04313-2 35562616 PMC9106608

[B11] Choi S.-C. Seo H. R. Cui L. H. Song M. H. Noh J. M. Kim K. S. (2021). Modeling hypoxic stress *in vitro* using human embryonic stem cells derived cardiomyocytes matured by FGF4 and ascorbic acid treatment. Cells 10 (10), 2741. 10.3390/cells10102741 34685725 PMC8534799

[B12] Cote-Vélez A. Cote-Velez A. Diaz-Gallardo M. Y. Perez-Monter C. Carreon-Rodriguez A. Charli J. L. (2005). Dexamethasone represses cAMP rapid upregulation of TRH gene transcription: Identification of a composite glucocorticoid response element and a cAMP response element in TRH promoter. J. Mol. Endocrinol. 34 (1), 177–197. 10.1677/jme.1.01634 15691887

[B13] Cruceanu C. Dony L. Krontira A. C. Fischer D. S. Roeh S. Di Giaimo R. (2021). Cell-type-specific impact of glucocorticoid receptor activation on the developing brain: A cerebral organoid study. Am. J. Psychiatry 179, 375–387. 10.1176/appi.ajp.2021.21010095 34698522

[B14] Cruceanu C. Dony L. Krontira A. C. Fischer D. S. Roeh S. Di Giaimo R. (2022). Cell-type-specific impact of glucocorticoid receptor activation on the developing brain: A cerebral organoid study. Am. J. Psychiatry 179 (5), 375–387. 10.1176/appi.ajp.2021.21010095 34698522

[B15] De Kloet E. Otte C. Kumsta R. Kok L. Hillegers M. H. J. Hasselmann H. (2016). Stress and depression: A crucial role of the mineralocorticoid receptor. J. Neuroendocrinol. 28 (8). 10.1111/jne.12379 26970338

[B16] De Kloet E. R. Meijer O. C. (2019). MR/GR signaling in the brain during the stress response. Aldosterone-Miner. Recept Cell Biol. Transl. Med. 10.5772/intechopen.87234

[B17] Di Lullo E. Kriegstein A. R. (2017). The use of brain organoids to investigate neural development and disease. Nat. Rev. Neurosci. 18 (10), 573–584. 10.1038/nrn.2017.107 28878372 PMC5667942

[B18] Finsterwald C. Alberini C. M. (2014). Stress and glucocorticoid receptor-dependent mechanisms in long-term memory: From adaptive responses to psychopathologies. Neurobiol. Learn. Mem 112, 17–29. 10.1016/j.nlm.2013.09.017 24113652 PMC3979509

[B19] Franklin T. B. Saab B. J. Mansuy I. M. (2012). Neural mechanisms of stress resilience and vulnerability. Neuron 75 (5), 747–761. 10.1016/j.neuron.2012.08.016 22958817

[B20] Garcia T. Y. Gutierrez M. Reynolds J. Lamba D. A. (2015). Modeling the dynamic AMD-associated chronic oxidative stress changes in human ESC and iPSC-derived RPE cells. Invest. Ophthalmol. Vis. Sci. 56 (12), 7480–7488. 10.1167/iovs.15-17251 26595608

[B21] Ghasemi M. Turnbull T. Sebastian S. Kempson I. (2021). The MTT assay: Utility, limitations, pitfalls, and interpretation in bulk and single-cell analysis. Int. J. Mol. Sci. 22 (23), 12827. 10.3390/ijms222312827 34884632 PMC8657538

[B22] Gordon A. Geschwind D. H. (2020). Human *in vitro* models for understanding mechanisms of autism spectrum disorder. Mol. Autism 11 (1), 26–18. 10.1186/s13229-020-00332-7 32299488 PMC7164291

[B23] Graudejus O. Ruben D. Wong P. Varghese N. Wagner S. Morrison B. (2018). “Bridging the gap between *in vivo* and *in vitro* research: Reproducing *in vitro* the mechanical and electrical environment of cells *in vivo* ,” in MEA Meeting 2018, 11th International Meeting on Substrate Integrated Microelectrode Arrays, Reutlingen, Germany, 04 Jul 2018-06 Jul 2018.

[B24] Heard K. J. Shokhirev M. N. Becronis C. Fredlender C. Zahid N. Le A. T. (2021). Chronic cortisol differentially impacts stem cell-derived astrocytes from major depressive disorder patients. Transl. Psychiatry 11 (1), 608–609. 10.1038/s41398-021-01733-9 34848679 PMC8632962

[B25] Hedegaard A. Monzon-Sandoval J. Newey S. E. Whiteley E. S. Webber C. Akerman C. J. (2020). Pro-maturational effects of human iPSC-derived cortical astrocytes upon iPSC-derived cortical neurons. Stem Cell Rep. 15 (1), 38–51. 10.1016/j.stemcr.2020.05.003 PMC7363746 32502466

[B26] Johnson E. O. Kamilaris T. C. Chrousos G. P. Gold P. W. (1992). Mechanisms of stress: A dynamic overview of hormonal and behavioral homeostasis. Neurosci. Biobehav. Rev. 16 (2), 115–130. 10.1016/s0149-7634(05)80175-7 1630726

[B27] Kim J. B. Ju J. Y. Kim T. Y. Yang B. H. Lee Y. S. Son H. (2004). Dexamethasone inhibits proliferation of adult hippocampal neurogenesis *in vivo* and *in vitro* . Brain Res. 1027 (1-2), 1–10. 10.1016/j.brainres.2004.07.093 15494151

[B28] Koning A.-S. C. Buurstede J. C. van Weert L. T. C. M. Meijer O. C. (2019). Glucocorticoid and mineralocorticoid receptors in the brain: A transcriptional perspective. J. Endocr. Soc. 3 (10), 1917–1930. 10.1210/js.2019-00158 31598572 PMC6777400

[B29] Krontira A. C. Cruceanu C. Binder E. B. (2020). Glucocorticoids as mediators of adverse outcomes of prenatal stress. Trends Neurosci. 43 (6), 394–405. 10.1016/j.tins.2020.03.008 32459992

[B30] Lieberman R. Kranzler H. R. Levine E. S. Covault J. (2017). Examining FKBP5 mRNA expression in human iPSC-derived neural cells. Psychiatry Res. 247, 172–181. 10.1016/j.psychres.2016.11.027 27915167 PMC5191911

[B31] Liu D. Nguyen T. T. L. Gao H. Huang H. Kim D. C. Sharp B. (2021). TCF7L2 lncRNA: A link between bipolar disorder and body mass index through glucocorticoid signaling. Mol. Psychiatry 26 (12), 7454–7464. 10.1038/s41380-021-01274-z 34535768 PMC8872993

[B32] Marchetto M. C. Gage F. H. (2012). Modeling brain disease in a dish: Really? Cell stem Cell 10 (6), 642–645. 10.1016/j.stem.2012.05.008 22704498

[B33] McEwen B. S. Nasca C. Gray J. D. (2016). Stress effects on neuronal structure: hippocampus, amygdala, and prefrontal cortex. Neuropsychopharmacology 41 (1), 3–23. 10.1038/npp.2015.171 26076834 PMC4677120

[B34] McEwen B. S. (2002). Sex, stress and the hippocampus: Allostasis, allostatic load and the aging process. Neurobiol. Aging 23 (5), 921–939. 10.1016/s0197-4580(02)00027-1 12392796

[B35] McEwen B. S. (1998). Stress, adaptation, and disease: Allostasis and allostatic load. Ann. N. Y. Acad. Sci. 840 (1), 33–44. 10.1111/j.1749-6632.1998.tb09546.x 9629234

[B36] McMaster A. Ray D. W. (2008). Drug insight: Selective agonists and antagonists of the glucocorticoid receptor. Nat. Clin. Pract. Endocrinol. Metab. 4 (2), 91–101. 10.1038/ncpendmet0745 18212811

[B37] Musazzi L. Tornese P. Sala N. Popoli M. (2017). Acute or chronic? A stressful question. Trends Neurosci. 40 (9), 525–535. 10.1016/j.tins.2017.07.002 28778394

[B38] Ninomiya E. Hattori T. Toyoda M. Umezawa A. Hamazaki T. Shintaku H. (2014). Glucocorticoids promote neural progenitor cell proliferation derived from human induced pluripotent stem cells. SpringerPlus 3 (1), 527. 10.1186/2193-1801-3-527 25279318 PMC4174547

[B39] Notaras M. Lodhi A. Barrio-Alonso E. Foord C. Rodrick T. Jones D. (2021). Neurodevelopmental signatures of narcotic and neuropsychiatric risk factors in 3D human-derived forebrain organoids. Mol. Psychiatry 26 (12), 7760–7783. 10.1038/s41380-021-01189-9 34158620 PMC8873021

[B40] Numakawa T. Kumamaru E. Adachi N. Yagasaki Y. Izumi A. Kunugi H. (2009). Glucocorticoid receptor interaction with TrkB promotes BDNF-triggered PLC-γ signaling for glutamate release via a glutamate transporter. Proc. Natl. Acad. Sci. U. S. A. 106 (2), 647–652. 10.1073/pnas.0800888106 19126684 PMC2626757

[B41] Nürnberg E. Horschitz S. Schloss P. Meyer-Lindenberg A. (2018). Basal glucocorticoid receptor activation induces proliferation and inhibits neuronal differentiation of human induced pluripotent stem cell-derived neuronal precursor cells. J. Steroid Biochem. Mol. Biol. 182, 119–126. 10.1016/j.jsbmb.2018.04.017 29751108

[B42] Odaka H. Adachi N. Numakawa T. (2017). Impact of glucocorticoid on neurogenesis. Neural Regen. Res. 12 (7), 1028–1035. 10.4103/1673-5374.211174 28852377 PMC5558474

[B43] Peng Q. Yan H. Wen Y. Lai C. Shi L. (2018). Association between NR3C1 rs41423247 polymorphism and depression: A PRISMA-compliant meta-analysis. Medicine 97 (39), e12541. 10.1097/MD.0000000000012541 30278546 PMC6181539

[B44] Popoli M. Yan Z. McEwen B. S. Sanacora G. (2012). The stressed synapse: The impact of stress and glucocorticoids on glutamate transmission. Nat. Rev. Neurosci. 13 (1), 22–37. 10.1038/nrn3138 PMC3645314 22127301

[B45] Provençal N. Arloth J. Cattaneo A. Anacker C. Cattane N. Wiechmann T. (2019). Glucocorticoid exposure during hippocampal neurogenesis primes future stress response by inducing changes in DNA methylation. Proc. Natl. Acad. Sci. U. S. A. 117, 23280–23285. 10.1073/pnas.1820842116 31399550 PMC7519233

[B46] Raciti M. Ong J. Weis L. Edoff K. Battagli C. Falk A. (2016). Glucocorticoids alter neuronal differentiation of human neuroepithelial-like cells by inducing long-lasting changes in the reactive oxygen species balance. Neuropharmacology 107, 422–431. 10.1016/j.neuropharm.2016.03.022 26992751

[B47] Regenmortel M. H. V. (2004). Reductionism and complexity in molecular biology: Scientists now have the tools to unravel biological complexity and overcome the limitations of reductionism. EMBO Rep. 5 (11), 1016–1020. 10.1038/sj.embor.7400284 15520799 PMC1299179

[B48] Richter-Levin G. Sandi C. (2021). Labels matter: Is it stress or is it trauma? Transl. Psychiatry 11 (1), 1–9. 10.1038/s41398-021-01514-4 34247187 PMC8272714

[B49] Sabbagh J. J. Cordova R. A. Zheng D. Criado-Marrero M. Lemus A. Li P. (2018). Targeting the FKBP51/GR/Hsp90 complex to identify functionally relevant treatments for depression and PTSD. ACS Chem. Biol. 13 (8), 2288–2299. 10.1021/acschembio.8b00454 29893552 PMC6126901

[B50] Sapolsky R. M. (2000). Glucocorticoids and hippocampal atrophy in neuropsychiatric disorders. Arch. Gen. Psychiatry 57 (10), 925–935. 10.1001/archpsyc.57.10.925 11015810

[B51] Sapolsky R. M. (1996). Stress, glucocorticoids, and damage to the nervous system: The current state of confusion. Stress 1 (1), 1–19. 10.3109/10253899609001092 9807058

[B52] Schoenfeld T. J. Cameron H. A. (2015). Adult neurogenesis and mental illness. Neuropsychopharmacology 40 (1), 113–128. 10.1038/npp.2014.230 25178407 PMC4262910

[B68] Seah C. Breen M. S. Rusielewicz T. Bader H. N. Xu C. Hunter C. J. (2021). Modeling gene x environment interactions in PTSD using glucocorticoidinduced transcriptomics in human neurons. Nat. Neurosci. 25, 1434. 10.1038/s41593-022-01161-y PMC9630117 36266471

[B53] Sheridan J. F. Padgett D. A. Avitsur R. Marucha P. T. (2004). Experimental models of stress and wound healing. World J. Surg. 28 (3), 327–330. 10.1007/s00268-003-7404-y 14961184

[B54] Shou Y. Liang F. Xu S. Li X. (2020). The application of brain organoids: From neuronal development to neurological diseases. Front. Cell Dev. Biol. 8, 579659. 10.3389/fcell.2020.579659 33195219 PMC7642488

[B55] Smoller J. W. (2016). The genetics of stress-related disorders: PTSD, depression, and anxiety disorders. Neuropsychopharmacology 41 (1), 297–319. 10.1038/npp.2015.266 26321314 PMC4677147

[B56] Sutanto W. De Kloet E. (1994). The use of various animal models in the study of stress and stress-related phenomena. Lab. Anim. 28 (4), 293–306. 10.1258/002367794780745092 7830368

[B57] Vadodaria K. C. Ji Y. Skime M. Paquola A. C. Nelson T. Hall-Flavin D. (2019). Altered serotonergic circuitry in SSRI-resistant major depressive disorder patient-derived neurons. Mol. Psychiatry 24 (6), 808–818. 10.1038/s41380-019-0377-5 30903001 PMC7409972

[B58] Vadodaria K. C. Ji Y. Skime M. Paquola A. Nelson T. Hall-Flavin D. (2019). Serotonin-induced hyperactivity in SSRI-resistant major depressive disorder patient-derived neurons. Mol. Psychiatry 24 (6), 795–807. 10.1038/s41380-019-0363-y 30700803

[B59] Vadodaria K. C. Ji Y. Skime M. Paquola A. Nelson T. Hall-Flavin D. (2019). Studying treatment resistance in depression using patient derived neurons *in vitro* . Mol. Psychiatry 24 (6), 775. 10.1038/s41380-019-0424-2

[B60] Volpato V. Webber C. (2020). Addressing variability in iPSC-derived models of human disease: Guidelines to promote reproducibility. Dis. Model. Mech. 13 (1), dmm042317. 10.1242/dmm.042317 31953356 PMC6994963

[B61] Wong P. Sze Y. Gray L. J. Chang C. C. R. Cai S. Zhang X. (2015). Early life environmental and pharmacological stressors result in persistent dysregulations of the serotonergic system. Front. Behav. Neurosci. 9, 94. 10.3389/fnbeh.2015.00094 25964750 PMC4410609

[B62] Xi G. Zhang X. Zhang L. Sui Y. Hui J. Liu S. (2011). Fluoxetine attenuates the inhibitory effect of glucocorticoid hormones on neurogenesis *in vitro* via a two-pore domain potassium channel, TREK-1. Psychopharmacology 214 (3), 747–759. 10.1007/s00213-010-2077-3 21069514

[B63] Xicoy H. Wieringa B. Martens G. J. (2017). The SH-SY5Y cell line in Parkinson’s disease research: A systematic review. Mol. Neurodegener. 12 (1), 10–11. 10.1186/s13024-017-0149-0 28118852 PMC5259880

[B64] Yehuda R. (2002). Post-traumatic stress disorder. N. Engl. J. Med. 346 (2), 108–114. 10.1056/NEJMra012941 11784878

[B65] Yeo Y. Tan J. B. L. Lim L. W. Tan K. O. Heng B. C. Lim W. L. (2019). Human embryonic stem cell-derived neural lineages as *in vitro* models for screening the neuroprotective properties of lignosus rhinocerus (cooke) ryvarden. Biomed. Res. Int. 2019, 3126376. 10.1155/2019/3126376 33204680 PMC7658738

[B66] Zhou M. Hoogenraad C. C. Joels M. Krugers H. J. (2012). Combined β-adrenergic and corticosteroid receptor activation regulates AMPA receptor function in hippocampal neurons. J. Psychopharmacol. 26 (4), 516–524. 10.1177/0269881111424930 21965192

[B67] Zhu L. J. Ni H. Y. Chen R. Chang L. Shi H. J. Qiu D. (2018). Hippocampal nuclear factor kappa B accounts for stress induced anxiety behaviors via enhancing neuronal nitric oxide synthase (nNOS)-carboxy-terminal PDZ ligand of nNOS-Dexras1 coupling. J. Neurochem. 146 (5), 598–612. 10.1111/jnc.14478 29858554

